# Impact of Adverse Childhood Experiences in Young Adults and Adults: A Systematic Literature Review

**DOI:** 10.3390/pediatric16020040

**Published:** 2024-06-07

**Authors:** Candy Silva, Patrícia Moreira, Diana Sá Moreira, Filipa Rafael, Anabela Rodrigues, Ângela Leite, Sílvia Lopes, Diana Moreira

**Affiliations:** 1 Centre for Philosophical and Humanistic Studies, Faculty of Philosophy and Social Sciences, Universidade Católica Portuguesa, 1649-023 Braga, Portugal; candysilva348@gmail.com (C.S.); patriciaigmoreira@gmail.com (P.M.); anabela.rodrigues@ucp.pt (A.R.); aleite@ucp.pt (Â.L.); 2 Institute of Psychology and Neuropsychology of Porto—IPNP Health, 4100-136 Porto, Portugal; diana.sa.moreira@gmail.com; 3 School of Education, The Polytechnic Institute of Oporto (IPP), 4200-465 Porto, Portugal; filipa.c.rafael@gmail.com; 4 CICPSI, Faculdade de Psicologia, Universidade de Lisboa, Alameda da Universidade, 1649-013 Lisboa, Portugal; 5 Centro de Solidariedade de Braga/Projecto Homem, R. do Alcaide 31, 4700-024 Braga, Portugal; 6 Observatory Permanent Violence and Crime (OPVC), FP-I3ID, University Fernando Pessoa, 4249-004 Porto, Portugal; 7 Laboratory of Neuropsychophysiology, Faculty of Psychology and Educational Sciences and CPUP—Center for Psychology at University of Porto, 4200-135 Porto, Portugal

**Keywords:** ACEs, adverse childhood experiences, offender, criminal, delinquent, traumatic events in childhood

## Abstract

Background: Investigations have shown the different impacts that ACEs have on an individual’s adult life, on both physical and mental health, but they have not yet shown the issue of the influence of ACEs on adults and young adults. Objective/Participants and Setting: This systematic review, performed according to the PRISMA norms and guidelines, intended to understand the most frequent outcomes of adverse childhood experiences in the life of young adults and adults. Methods: Studies were identified through multiple literature search databases at EBSCOhost, Web of Science, and PubMed April 2023, and a total of 279 studies, published between 1999 and 2002, were excluded, 256 because of multiple factors: being duplicates, showing statistical analysis with correlations only, being systematic reviews or case studies, comprising individuals under the age of 18, and not meeting the intended theme; ultimately, we selected for the review a total of 23 studies. Results and Conclusions: The impacts of the various articles are subdivided into three main themes: antisocial and criminal behaviour; sexual Behaviour and intimate partner violence; and attachment, quality of life, and therapeutic alliance.

## 1. Introduction

Studies have shown the importance of looking into the relationship between multiple forms of victimisation, taking into account the characteristics of disclosure connected to the risk of revictimisation, and the significance of the support received [1]. In fact, maltreatment can happen through the intentional use of force or power, either in its actual act or in a threat, against oneself, another individual or a group or community, the outcome of which is (or has the possibility of being) an injury, death, psychological deterioration, affected development, or deprivation [2]. Moreover, it can be perpetrated through various ways and means, such as domestic alcohol or substance abuse, parental divorce or separation, community violence, bullying, exposure to family violence, and family involvement in criminal activities. However, the World Health Organisation (WHO) distinguishes four natures of violence against children: physical, sexual, emotional, or psychological abuse and neglect [3].

Therefore, child physical abuse (CPA) is defined by the WHO as any intentional action against the child by their parents/guardians that threatens/harms (or is likely to harm) their physical integrity [4]. This type of violence can cause several injuries, such as scratches, abdominal injuries, fractures, contusions, and head trauma [5].

Emotional abuse refers to being humiliated, rejected, deteriorated, isolated, and terrified, as well as rejected emotional responsiveness. This includes, for example, scolding harshly, despising the child’s abilities and achievements, and bullying [6]. 

Subsequently, child sexual abuse (CSA) corresponds to any act with a child that is aimed at the sexual gratification of the adult or another significantly older and more powerful child. This type of violence includes touching, oral and genital contact, rape, genital or anal penetration, exhibitionism, voyeurism, and/or pornographic exposure, which is neither consented to nor understood by the child [7] and usually occurs in congruence with other types of CSAs [8]. Childhood victimisation of sexual violence is estimated to be associated with an increased risk of engaging in sexual violence [9]. Most of the time, these events remain silent within families, where these are one of the most important influences on a child’s development, so it is extremely important to find out if it is a home that provides support and love or if it is characterised by conflict and violence [2]. This is because the course of a given individual’s life is influenced by all the social factors with which they come into contact, i.e., all past events may affect future prospects, either positively or negatively [10]. Understanding of the harms associated with adverse childhood experiences (ACEs) has developed considerably over the past two decades. Based on a spread of literature about the prevalence of ACEs and their link to lifelong health problems [11]). One notable study by Medved et al. (2020) emphasises the potential of the axiological approach in preventing ACEs by highlighting the significance of socio-cultural practices in educational settings and families [12]. This research underscores the importance of fostering positive interactions and support systems to prevent ACEs. Moreover, the research by Rodriguez et al. (2021) has explored the relationships among early adversity, positive interactions, and mental health in young adults, highlighting the significant impact of adverse experiences on mental well-being and the potential buffering effect of positive human and animal interactions. This study underscores the importance of fostering supportive environments for young adults exposed to ACEs to promote mental health resilience [13]. Also, ACEs have been linked with the motive of early death, mental illness, addiction, and poor health [14].

In fact, a life history characterised by the experience of ACEs may reflect negatively on the victims’ development process. For example, it may result in lack of interest, dispersion, and inattention in class; learning difficulties; and low performance in adolescent victims of abuse [15]. It may eventually lead them to several future problems, such as social isolation, alcoholism, and numerous psychopathologies, including suicidal behaviour [16]. Furthermore, people who suffered from childhood maltreatment, showed more aggression [17]; despite this, all ACEs involve aggressive and/or violent behaviour in adult individuals [9].

Caregiver abuse is correlated with an increased likelihood of developing antisocial personality disorder [18]. Furthermore, when ACE types are analysed, it is found that abuse affects most personality traits [19]. 

Moreover, ACEs have long-lasting negative effects on cognitive performance and brain development. Neuropsychological or cognitive deficits are related to an increased risk of longitudinal criminal offending, recalling that ACEs are capable of damaging neurocognitive functions during key developmental stages [20].

In fact, the presence of ACEs has been shown to predict mental disorders and mental health problems and increase suicide levels even after controlling for sociodemographic factors and other forms of adverse experiences in childhood and adulthood [7]. ACEs had distinct associations with the three offences by racial and ethnic group. African Americans with ACEs were 39% less likely to commit homicide, and Hispanics with four adverse childhood experiences were 100% more likely to commit homicide. Moreover, White people with five or six adverse childhood experiences were 68% and 85% less likely to commit homicide, respectively [21]). Adverse childhood experiences (ACEs) have been shown to have distinct associations with different types of offenses across racial and ethnic groups. Research by Fagan and Novak (2017) highlighted that the impact of ACEs on depression, illegal drug use, and delinquency varied by race, with ACEs having stronger effects on White people compared to Black people [22]. Similarly, Zhang and Monnat (2022) found that racial/ethnic differences exist in the clusters of ACEs experienced by adolescents, with a small share of adolescents in all racial/ethnic groups being in a “high global adversity” class characterised by exposure to multiple ACEs [23]. Moreover, Gu et al. (2022) suggested that the higher frequency of ACEs among Hispanic and Black youth may lead to potential differences in the types of ACEs experienced and the pathways through which these experiences influence health among different racial and ethnic groups. These findings indicate that the effects of ACEs on various outcomes, including delinquency and mental health, can vary across racial and ethnic lines [24]. Childhood trauma is marked in a sizable fraction of inmates, with 37% experiencing physical neglect and 68% emotional neglect, with dissociative identity disorder and development of aggressive and violent behaviours being visible. In fact, it is relevant to mention that existing reports demonstrate substantially higher rates of ACEs among incarcerated subjects compared to the general population [25]. As an example, female inmates reveal more exposure to ACEs, showing higher levels of negative emotional states, such as depression, anxiety, and stress, in analogy to female inmates who did not disclose. Across all categories, women exposed to ACEs revealed substantially higher levels of unfavourable emotional states and lower levels of social support in every proportion) [25]. Indeed, these individuals have a childhood delineated by domestic abuse, having experienced physical and psychological aggression, exposure to domestic violence, neglect, lack of affection, feelings of indifference, and parental hostility. This creates feelings of abandonment, insecurity, and loneliness, also affecting self-esteem and hindering their ability to relate to other people, thus interfering with several parameters of their adult life [10]. A study that examined the associations between ACEs, paraphilias, and serious criminal violence among federal sex offenders found that offenders had nearly five paraphilias on average; the most common were paedophilia (57%), pornography addiction (43%), unspecified paraphilia (35%), exhibitionism (26%), and voyeurism (21%) [26].

In fact, to try to explain all the consequences of ACEs in the adult lives of victims, we address several theories. In fact, social pathways like education, work, and family have an effect in developmental patterns and life trajectories, according to the Life Course Theory [27]. Low parental supervision, crime, and neighbourhood victimisation are not exclusively factors that create circumstances conducive to crime but are also developmental risk factors that shape children’s actions and future behaviour [28]. Subsequently, Social Learning Theory helps us understand how the ACEs have a bigger influence than in adulthood, since these abuse behaviours in childhood are learned and later manifested in adolescence, continuing until the adult life [29].

On the other hand, Strain Theory explains delinquency in terms of the individual’s social relationships. It focuses explicitly on negative relationships with others: relationships in which the individual is not treated as they want to be treated, relationships in which others prevent the individual from achieving positively valued goals, and relationships in which others present the individual with harmful or negative stimuli. Strain Theory argues that adolescents are pressured into delinquency by negative affective states—especially anger and emotions that result from negative affective states. This creates pressure for corrective action and can lead adolescents to make use of illegitimate channels [30].

Another theory is the Social Control Theory, which states that the perpetuation of criminal behaviour can be found in the ties that people form with pro-social values, pro-social people, and pro-social institutions. These bonds ultimately control our behaviour when we are tempted to engage in criminal or deviant acts. In fact, pro-social attitudes restrict people from committing the crimes they would otherwise have without such social ties, which indirectly control our behaviour [31].

According to the Psychoanalytic Theory, early childhood experiences influence have a great influence in personality development and psychological well-being, meaning that a deviant behaviour can be a result of the parental behaviour that occurred during the child’s early development [32].

The Labelling Theory explains that even if an individual is known to have been abused in childhood, they are eventually labelled by the system as an offender. Often, this labelling leads to children being removed from the family environment, which can promote the adoption of criminal behaviour in the long term. However, the victim may turn to crime to escape the traumatisation that characterises abuse, because of association with the wrong set of peers, because of diminished self-esteem, as a symbol of revenge against the perpetrator, or as a post-traumatic adaptive response [33].

Children create thoughts and behaviour patterns according to their relationship with their primary caregiver. When the relationship with the primary caregiver is characterised by being abusive, children create an insecure attachment, which correlates positively in adolescence and adulthood with sexual offense. When children create this insecure attachment, it leads to psychological and social deficits associated with criminal necessities, with are risk factors linked to deviant behaviour; the more risk factors someone has, more likely they are to commit a crime [34].

Finally, Trauma Acquisition Theory suggests that trauma information processing highlights the limbic system as the primary system for encoding related information, which interferes with the process of retrieval and recall. If the limbic system is overwhelmed by incoming information, there is an initial transformation response, which, if it fails to manage and respond to the information, becomes the survival response of numbing and dissociation [35].

Having said this and considering the severity and dimension of this issue, this article focused on systematically summarising the specificities of the impact of adverse childhood experiences on adults and young adults, reviewing its components and describing the results present in the literature. Studying the impact of ACEs is crucial due to their profound effects on various aspects of individuals’ lives. Research has shown that ACEs can have lasting consequences on mental health, behaviour, and overall well-being [36]. Understanding the intergenerational impact of ACEs and incorporating a resilience framework into interventions are essential steps in addressing the effects of ACEs [37]. Investigating the impact of ACEs on different presentations, from normality to psychopathology, provides valuable information on the diverse outcomes associated with ACEs [38]. Preventing ACEs should be a priority, as cumulative adversity affects children from different racial/ethnic and income groups. By examining the dose–response effect of ACEs and identifying risk and protective factors, public health efforts can be better informed to prevent ACEs and promote flourishing in children. Preventing ACEs and child maltreatment has been deemed a priority by various international child welfare organisations, with various prevention and mitigation models proposed [39]. Policies and practices that address intergenerational impacts of historical legacies of oppression are needed [40]. It is critical to prevent ACEs and build resilience in individuals affected by ACEs [41].

In fact, a systematic review focusing on the impact of ACEs in adults and young adults is crucial for the community for several reasons. Firstly, such a review can provide a comprehensive understanding of how ACEs during childhood can manifest in health outcomes and behaviours in adulthood, shedding light on the long-term consequences of early life adversity. By synthesising the existing literature on the relationship between ACEs and health outcomes in young adults, a systematic review can offer valuable insights into the mechanisms through which ACEs influence coping strategies and overall well-being [42]. Understanding these pathways is crucial for developing targeted interventions to support individuals who have experienced ACEs and to prevent the negative outcomes associated with such experiences. Moreover, a systematic review on the impact of ACEs in adults and young adults can inform policymakers, healthcare providers, and community organisations about the prevalence and implications of ACEs in different populations. By examining patterns of ACEs and their association with various health outcomes, such a review can guide the development of evidence-based interventions and policies aimed at mitigating the effects of ACEs and promoting resilience among affected individuals [43]. This knowledge is essential for creating trauma-informed communities that can provide appropriate support and resources for individuals who have experienced ACEs. Additionally, a systematic review on ACEs in adults and young adults can highlight the need for early identification and intervention strategies to address the consequences of childhood adversity [44]. Furthermore, a systematic review can contribute to the growing body of knowledge on the intergenerational transmission of trauma and adversity, emphasising the importance of breaking the cycle of ACEs within families and communities. By examining the impact of ACEs on caregivers and subsequent generations, such a review can inform interventions that aim to promote resilience and positive outcomes for children and families affected by ACEs [45]. This knowledge is essential for developing holistic approaches to addressing the complex and interconnected challenges associated with ACEs.

As matter of fact, a systematic review of the impact of ACEs on adults and young adults is crucial for several reasons. Firstly, such a review would provide a comprehensive understanding of how ACEs influence various aspects of individuals’ lives and their transition into adulthood. By synthesising existing literature, a systematic review can highlight the associations between ACEs and their consequences. Moreover, understanding the relationship between ACEs and health outcomes in young adults is essential for developing targeted interventions and prevention strategies. By conducting a systematic review, researchers can identify gaps in knowledge, explore potential mechanisms underlying these associations, and inform the development of evidence-based interventions to mitigate the negative impact of ACEs on health outcomes in young adults. Additionally, a systematic review can shed light on the intergenerational transmission of ACEs and their effects on the next generation. By examining the impact of ACEs on young adults who may become parents themselves, researchers can better understand how early life adversity influences parenting practices, family dynamics, and the well-being of future generations.

Considering that there are multiple studies regarding the impacts of ACEs in adults and young adults, they specify some topic, for example, ACEs and adult health outcomes, young people attending clinical and healthcare settings, neurocognition in borderline personality disorder, related outcomes among adults experiencing homelessness, chronic lung diseases in adulthood, childhood obesity, substance use in young adults [46,47,48,49,50,51,52], and others; however we did not find any systematic review as embracing as ours. So, this literature review aims to answer the following question: “What is the impact of adverse childhood experiences on adults and young adults?”.

## 2. Materials and Methods

Studies were identified through multiple literature search databases at EBSCOhost, Web of Science, and PubMed. The search expression base terms were AB (“traumatic events in childhood” OR negative events in childhood OR adverse childhood experience* OR ACE) AND TI (offender* OR criminal* OR prisoner OR felons OR convictions OR inmate* OR delinquent*). Furthermore, the search was not restricted to geographic, temporal, or linguistic factors.

Study selection and data extraction were performed by two independent reviewers, as proposed by the Cochrane Collaboration [53]. The rate of agreement in the selection process was assessed by Cohen’s kappa, with a percentage of agreement of 82.53%. Disagreements between the reviewers were discussed, and consensus was reached.

Cross-sectional and longitudinal empirical studies, with individuals older than 18 years, and that presented statistical regression were included, while studies with individuals younger than 18 years, systematic reviews, case studies, and those that performed statistical analysis using only correlations were excluded, because correlation indicates a relationship between two variables but does not prove that one variable causes the other.

A total of 279 studies published between 1999 and 2023 were identified from all databases and search methods. Of these, 143 studies were excluded because they were duplicates, 170 showed statistical analysis with correlations only, 70 were either systematic reviews or case studies, and 12 comprised individuals under the age of 18. Of the 28 studies remaining from the integrative reading, five were excluded because they did not meet the intended theme. Thus, a total of 23 studies were selected for review.

### Reliability and Validity

**Figure 1:**
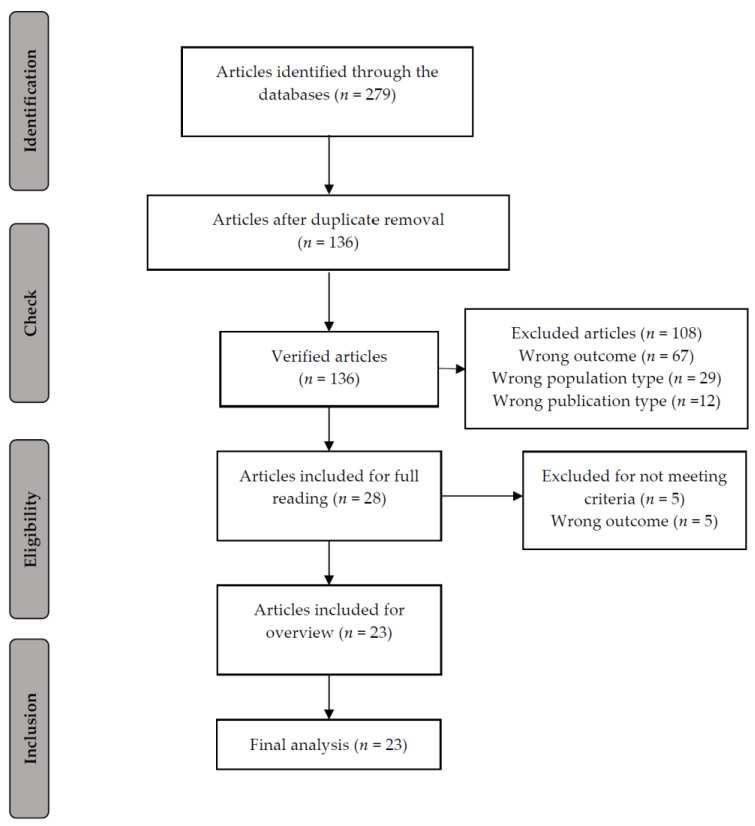
Flow diagram.

Systematic reviews play a key role in the synthesis of evidence. Therefore, to ensure the reliability and validity of systematic reviews, we implemented strict control procedures to mitigate bias. In this way, we used established guidelines, such as the Preferred Reporting Items for Systematic Reviews and Meta-Analyses (PRISMA) checklist, to guide the review process and reduce bias [54]. The PRISMA flowchart (Figure 1) and checklist provide a structured framework that allows the methods, results, and conclusions to be presented transparently, thus reinforcing the quality and credibility of the systematic review. In addition, defining pre-specified objectives and research questions before starting the review is crucial for reducing bias [55]. By clearly outlining the research objectives and methods in advance, we avoid post hoc changes that could introduce bias into the review process. To combat bias related to changes in the specifications of the results during the review, we share the entire process of the systematic review [56]. This practice promotes transparency and accountability, mitigating the risk of selective reporting of results and maintaining the integrity of the review results. In fact, by adhering to the established guidelines and communicating their methods in a transparent manner, we increase the credibility, reliability, and validity of the results, thus contributing significantly to evidence-based decision making.

## 3. Results

A summary of the characteristics of the included studies is presented in Table 1.

Based on the characteristics of the reviewed studies, this section presents the main findings emerging from the investigation of various groups of individuals who can be characterised as individuals who have suffered adverse childhood experiences.

### 3.1. Antisocial and Criminal Behavior

#### 3.1.1. Young Adults

Specific adverse experiences during childhood are linked to different dimensions of psychopathic traits. Physical abuse is associated with the behavioural dimension of psychopathy, while emotional abuse is linked with the callous/unemotional traits of psychopathy. Therefore, the diversity of adverse childhood experiences is a strong predictor of juvenile delinquency and adult crime. Additionally, sexual abuse is the type of maltreatment most associated with different dimensions of youth psychopathy, including traits of manipulation, dishonest charm, callousness, and unemotionality. Notably, abuse and neglect have a negative correlation with altruistic attitudes, suggesting that children who experience maltreatment are less likely to develop prosocial skills compared to those who experience support and affection without violence [57].

Country of residence was found to be significantly associated with self-reported criminal variety, at 16.60%, particularly in subgroups such as males and those living in the Human Development Index top-tier countries. The four ACEs of physical abuse, sexual abuse, physical neglect, and household substance abuse during the first 18 years of life were significant predictors of criminal variety among young adults aged 18–20 in both HDI top and bottom tier countries (Table 2) [58].

Significant relationships between early adversity and negative outcomes such as juvenile justice involvement, criminal persistence, and psychosocial problems during early adulthood are shown by this study. Juvenile justice involvement, Exp(*β*) = 4.51 and *p* = 0.006, and criminal persistence, Exp(*β*) = 3.98 and *p* = 0.040, are strongly predicted by sexual abuse, while emotional maltreatment, *β* = 0.22 and *p* = 0.064, and mental illness in the household, *β* = 0.28 and *p* = 0.010, predict psychosocial problems. This highlights the importance of screening for and preventing severe adversity in childhood and adolescence to mitigate the risk of negative outcomes in adulthood [59].

#### 3.1.2. Adults

Adults who have been exposed to complex trauma during childhood often exhibit emotional dysregulation and self-regulation deficits, which can lead to poor decision making and externalising behavioural problems. Consequently, childhood trauma can significantly hinder the development of executive function, thus increasing the likelihood of engaging in violent, aggressive, or criminal behaviour later in life, ORs = 4.54–10.53. Women who have experienced ACEs are at a significantly higher risk for engaging in violent behaviour, which indicates a clear and positive association between ACEs and violent criminal behaviour, men: ORs = 4.54–8.41; women: ORs = 7.27–10.53 [60].

A study conducted by Hilton and collaborators (2019) found that ACEs were associated with criminal propensity in all models, except institutional assaults among all offenders. However, among intimate partner violence (IPV) offenders, ACEs were only linked to the actual risk of violent recidivism, which could be partly explained by the Violence Risk Appraisal Guide (VRAG) item of marital history. This provides limited support for the relationship between ACEs and criminal propensity among IPV offenders, possibly due to the small sample size and restricted range of ACE scores. Furthermore, the study found no significant effect of intimate partner history on ACEs or criminal propensity measures. The only exception was a lower risk of violent recidivism associated with having an intimate relationship history, which could be partly explained by the VRAG item of marital history [61].

Moreover, according to the study by James et al. (2020), childhood maltreatment during the early years (0–12 years) is linked to sexual homicide using reactive aggression, ORs = 0.74. Additionally, exposure to inadequate parental behaviour is particularly detrimental in the development of reactive aggression, exacerbating the prediction of reactive aggression in sexual murderers, ORs = 0.68. However, victimisation in adolescence has not been associated with sexual homicide. Furthermore, the study revealed that impulsive or anger-driven homicides were observed in the sample [62].

Also, a relationship between variability in violence among violent offenders and diagnoses of adult ASPD and childhood conduct disorder was found in a study conducted by Hill and Nathan (2008), highlighting the importance of considering these factors when addressing violent behaviour. The study also found that interparental violence was strongly associated with both social violence, but only social violence was associated with reports of childhood conduct disorder and adult ASPD, indicating the need for targeted prevention strategies (Table 2) [63].

Cumulative adversity, like the ACE score, was a significant predictor for all outcomes in the study, according to Stinson et al. (2016), highlighting the impact of multiple adverse experiences on development. Additionally, foster care placements have the potential to affect a child’s development and attachment to caregivers, with individual differences and other associated factors playing a role. Therefore, early trauma and foster care placement were significant contributors to negative outcomes examined in the study, emphasising the importance of addressing these issues in efforts to promote positive development [64].

Childhood adversity increases the risk of criminal behaviour problems in adulthood for sex offenders, β = 0.107, Wald = 3.919, Exp(B) = 1.112, significance = 0.048, and R2 = 0.013, according to Levenson and Socia (2015). These offenders had higher rates of early adversity in all categories, indicating exposure to various childhood maltreatment and chaotic households. Higher ACE scores were associated with a greater assortment of arrest items, suggesting that early trauma accumulation increased the likelihood of versatile criminal behaviour. Furthermore, CSA, emotional neglect, and domestic violence in the childhood home were significant predictors of the total number of sex crime arrests, but not for non-sexual arrests, total arrests, or criminal versatility, related to the likelihood of versatile criminal behaviour. The largest effect of the ACE score on the arrest scale item was for non-sexual assault. Lower CSA, EN, and domestic violence in the childhood home were predictors of the total number of sex crime arrests, but not for non-sex arrests, total arrests, or criminal versatility (Table 3) [65].

In each follow-up assessment, the study discovered a stronger connection between cumulative ACE score and victimisation, AOR = 1.27, than CJI, AOR = 1.58. This implies that individuals with higher ACE scores are more likely to become victims rather than be involved in the criminal justice system. Moreover, regardless of the participants’ study arm or other confounding factors, the study found that the connection between cumulative ACE score and both victimisation and criminal justice remained significant in all four follow-up time points. However, the cumulative ACE score did not affect the intervention’s effects (housing first versus treatment as usual) on the likelihood of experiencing either outcome, indicating that the intervention’s effectiveness did not vary based on the participants’ cumulative ACE scores throughout the study, HF versus TAU: Exp(B) = 1.04 and *p* = 0.611 [66].

While the sum of ACEs does not have a significant association with symptoms of ASPD, a significant association was found with the disorder’s diagnostic history, which remains significant even after controlling for other covariates related to childhood psychopathology, substance use, and criminal career. Epidemiological research shows that ACEs are linked to the early family environments of criminal offenders, and there is considerable variability in the familial progression of ASPD [18].

Also, according to Koolen and Vos (2022), a remarkable result was the absence of significant correlations between ACEs and PD pathology. While mode domains explain part of the variance between ACEs and all three PDs, certain ACE categories account for a small portion of the variance for BPD and NPD. These findings suggest that trauma has little or no direct effect on PD pathology, in line with the notion that the relationship between ACEs and antisocial behaviour, PD pathology, or criminal behaviour is multifaceted [67].

Lastly a study conducted by Jones et al. (2020) indicates that the social functioning of a young adult group with multiple problems is influenced by both criminal history and ACEs, but to varying degrees. These factors have different levels of importance when it comes to social functioning [68].

### 3.2. Sexual Behavior and Intimate Partner Violence

#### 3.2.1. Young Adults

Boys who experienced more CPA were indeed more likely to perpetrate psychological violence against their partners, χ^2^ = 4.62, *p* = 0.032, and use multiple forms of violence, χ^2^ = 4.14, *p* = 0.042. Men who experienced more CPA were at an increased risk of sexual and psychological victimisation by their intimate partners, χ^2^ = 6.04, *p* = 0.014, and were more likely to report polyvictimisation. Similarly, when experienced more, CSA was more likely to perpetrate physical and sexual violence against individuals’ partners and the use of multiple forms of violence, where alcohol use was found to be a mediator in the relationship. Finally, men with more CEA experiences were more likely to experience physical and psychological victimisation in their intimate relationships. Moreover, they were more likely to report clinically depressive symptoms in early adulthood [69].

#### 3.2.2. Adults

In fact, the presence of ACEs in the development of individuals implies future intimate partner violence and sexual behaviour problems; having said that, considering clinical and criminological factors, it is possible to establish a connection between CSA and future sexual offending [26].

Moreover, childhood victimisation contributes to the development of criminogenic cognitions, antisocial behaviours, and sexual interests in children. Also, childhood abuse is a significant predictor of offence-supportive cognitions, substance abuse, and youth engagement in sexual offending among individuals involved in online sexual offending during adulthood. Then ACEs imply more sexual interest in children and a sense of loneliness, χ^2^ = 4.16, *p* = 0.041, although ACEs increase the risk of victimisation and perpetration in adulthood but do not determine it [70].

Additionally, ACEs such as CSA and neglect in women increase the likelihood of experiencing multiple forms of IPV, adult physical, sexual, and psychological abuse, are more likely to report adult sexual abuse and predict severe physical injury in adult abusive relationships. Women who have experienced five or more ACEs are at a higher risk of reporting adult physical, sexual, and psychological abuse than those with fewer ACEs (four or fewer), IRR = 1.354, *p* ≤ 0.001. Additionally, there is a link between ACEs, IPV, and women’s offending and incarceration, indicating that ACEs and IPV are significant risk factors for women’s involvement in the criminal justice system [71].

**Figure 2:**
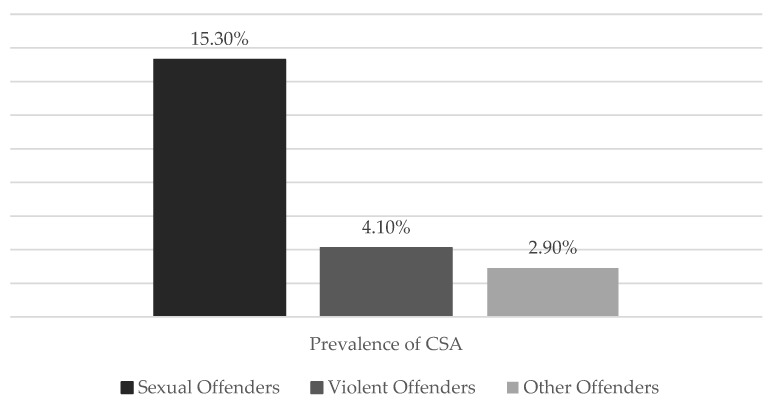
Prevalence of child sexual abuse in different types of offenders.

Lastly, the exposure to PA during childhood only and PA both in adulthood and childhood was associated with relative risk of violent crime. Also, CPA was associated with violent crime perpetration, but not sexual crime perpetration. In fact, the prevalence of exposures to CSA was more than three times greater among people convicted of sexual offenses compared to those convicted of violent only and more than five times greater than the prevalence of sexual abuse compared to those convicted of all other crimes (Figure 2). In the end, it was found that a high accumulation of ACEs (five or more) can imply the perpetration of IPV [9].

#### 3.2.3. Adults and Young Adults

Effectively, CEA, CSA, and family dysfunction are risk factors for paraphilias. CEA and family dysfunction were found to be common developmental risk factors for paedophilia, exhibitionism, rape, or multiple paraphilias, with emotional abuse contributing more significantly than family dysfunction. CSA was identified as a specific developmental risk factor for paedophilia [72].

### 3.3. Attachment, Quality of Life, and Therapy Alliance

#### 3.3.1. Young Adults

Regarding quality of life (QoL), childhood or adolescent maltreatment is linked to a decreased likelihood of possessing altruistic attitudes and an increased likelihood of exhibiting psychopathic traits in young adults. The impact of ACEs on QoL during young adulthood is significant; specifically, multiple ACEs experienced early in life were shown to be a strong predictor of QoL, even when accounting for criminal history. On the other hand, a lower number of ACEs was associated with better QoL outcomes. Therefore, the study results indicate that these factors have different levels of importance when it comes to social functioning [73].

#### 3.3.2. Adults

Referring to attachment styles, formerly incarcerated Black and Latino adult men are at a high risk of experiencing disrupted attachments, as per the implication of this study. The higher the number of ACEs an individual faces, the more probable it is for them to develop anxious and avoidant insecure attachment styles in adulthood. Consequently, childhood trauma is a significant predictor of insecure attachment patterns in formerly incarcerated Black and Latino men [74].

In fact, the relationship between adverse childhood experiences and the therapeutic alliance in male forensic patients with cluster B personality disorders is essential. While physical, sexual, and emotional abuse were not predictive of therapy alliance, childhood neglect had a negative effect on it. This is significant because therapy alliance is related to treatment outcome, recidivism, and adherence to probation conditions, and childhood neglect is prevalent in offender populations [75].

Lastly, referencing the general consequences of ACEs in adulthood, Moore and Tatman (2016) state that the ACE scale has been linked to a variety of negative outcomes in adulthood. For instance, studies have shown that higher ACE scores are predictive of future incarceration, recidivism, violent behaviour, and substance abuse. Furthermore, increased ACE scores have been associated with poor physical and mental health, chronic disease, premature mortality, and functional limitations. In fact, higher ACE scores are associated with increased scores on the LSI-R, β = 0.245, *p* < 0.01. Moreover, the social functioning of a young adult group with multiple problems was found to be influenced by both criminal history and ACEs, but to varying degrees [76].

## 4. Discussion

### 4.1. General Impact

Studies show that adverse childhood experiences have several impacts on the individual’s adult life, both in terms of physical and mental health. However, no systematic reviews have been found regarding the influence of ACEs on adults and young adults. Therefore, this review aims to understand the different influences that ACEs have on individuals who suffer from them to answer the following research question: What is the impact of ACEs on the lives of adults and young adults?

To gather more knowledge on this question, a systematic literature review was conducted following PRISMA guidelines, including the use of two independent researchers on selecting the studies for review. In fact, it is extremely important to address this topic, because the course of a given individual’s life is influenced by all the social factors with which they have contact, i.e., all past events may affect future perspectives, either positively or negatively [10].

Individuals who have experienced ACEs during childhood show feelings like abandonment, insecurity, and solitude, affecting their self-esteem and the ability to socialise with others [10]. In addition, studies addressing antisocial and criminal behaviour state that individuals who have complex trauma experiences exhibit emotional dysregulation and self-regulation deficits result in having poor decision making and externalising behaviour problems, as well as also exhibiting violent and aggressive behaviour [60]; adding to that, those who have had ACEs developed anxious and avoidant insecure attachment styles in adulthood [74], and childhood neglect has shown a negative effect in therapeutic alliance [75], which can be explained on the basis of Attachment Theory, because children develop beliefs and behavioural patterns based on the relationship they have with their primary caregiver, so if the relationship with attachment figures is an abusive one, insecure attachment will develop, which correlates positively with offending in adolescence and adulthood. In fact, abused children tend to form insecure attachments that lead to psychological and social deficits consistent with criminal needs.

### 4.2. Personality Traits and Psychopathy

In fact, childhood physical abuse influences the behavioural dimension of psychopathic traits; childhood emotional abuse does the same to callous/unemotional traits of psychopathy; and childhood sexual abuse influences youth psychopathic traits, like manipulation, dishonest charm, callousness, and unemotionality. In sum, ACEs are a strong predictor of juvenile delinquency and adult crime [57]. Furthermore, studies related to sexual behaviour and intimate partner violence state that it is possible to establish a connection between CSA and future sexual offending [26]. Besides CEA, CSA and family dysfunction are risk factors for paraphilias, paedophilia, exhibitionism, and rape, with emotional abuse contributing more significantly [72]. These facts can be accounted for by the Life Course Theory, which implies that social pathways such as education, work, and family act on developmental patterns and life trajectories, e.g., low parental supervision, abuse, crime, and neighbourhood victimisation are developmental risk factors that shape children’s actions and future behaviour.

Also, regarding the association of paraphilias with CSA, this can be explained by Social Learning Theory, which provides a robust framework for understanding why exposure to ACEs would be more influential than exposures during adulthood, suggesting that abusive behaviours are learned during childhood and manifested as aggression starting in adolescence and persisting into adulthood. Additionally, abuse has a strong effect on violent and aggressive behaviours in adults [77]. In fact, abused children may internalise these behaviours to believe that aggression is an acceptable form of interpersonal behaviour. This theory also explains that CPA is associated with an increased risk of violent crime and that CSA is associated with an increased risk of sexual offense. Furthermore, ACEs are linked to a decreased likelihood of possessing altruistic attitudes and an increased likelihood of exhibiting psychopathic traits in young adults [73].

### 4.3. Aggression

Besides this, exposure to inadequate parental behaviour provokes the development of reactive aggression [62], and interparental violence is associated with social violence and IPV [63]. These facts are related to the Psychoanalytic Theory that affirms that deviant behaviour results from parental behaviour during the child’s early development. Also, individuals with higher ACEs scores are more likely to become victims rather than to be involved in the criminal justice system [66]. This can be explained by Social Control Theory, which states that the perpetuation of criminal behaviour can be found in the bonds that people form with pro-social values, pro-social people, and pro-social institutions, bonds that control our behaviour.

In addition, ACEs are predictive of future incarceration, recidivism, violent behaviour, substance abuse, poor physical and mental health, chronic diseases, premature mortality, and functional limitations [76]. All of this relates to the Labelling Theory, which states that even if it is known that the individual was abused in childhood, they end up being labelled by the system as an offender. Moreover, the victim may resort to crime to escape the traumatisation that characterises the abuse. Often, this labelling leads to children being removed from the family environment, which can promote the adoption of criminal behaviour in the long term. So, being spanked as a child was strongly connected with every self-reported mental health outcome [78]. Besides this, more robust social bonds did not lower the harmful effects of exposure to many types of ACEs on relapse [79].

In closing, it is shown that numerous ACEs, including emotional dysregulation, self-regulation deficits, childhood neglect, and multiple forms of abuse, have been associated with a span of negative outcomes in adulthood, such as poor decision making, externalising behaviour problems, and aggressive behaviour. Besides being linked to violent crime, they are also connected to an increased risk of developing psychopathic traits, engaging in sexual offending, IPV, future incarceration, violent behaviour, and poor physical and mental health outcomes.

### 4.4. Limitations

This systematic review has some limitations. As with most systematic reviews, there is a risk of reporting bias as only studies published in identifiable sources were included, even though we did not make restrictions regarding geographical and linguistic criteria. Nevertheless, this review contributes to the analysis of the impact of ACEs on the lives of adults and young adults. A suggestion for future studies would be to conduct meta-analytic approaches on the topic, as they may be feasible soon, and as the data set on the impact of ACEs is still growing. In summary, there is a broad consensus regarding the impacts; however, they vary depending on the specifics of the studies reviewed.

## 5. Conclusions

This literature review aimed to understand the different influences that ACEs have on individuals who suffer from them. ACEs have been associated with a span of negative outcomes in adulthood such as poor decision making, externalising behaviour problems, and aggressive behaviour. They can also be linked to violent crime, and they are also connected to an increased risk of developing psychopathic traits, as well as engaging in sexual offending, IPV, future incarceration, violent behaviour, and poor physical and mental health outcomes. Furthermore, ACEs are also predictive of future incarceration, recidivism, violent behaviour, substance abuse, poor physical and mental health, chronic diseases, premature mortality, and functional limitations.

## Figures and Tables

**Table 1:** Summary of included studies.

Study	Main Objective	Origin Country	Participants (Age in Years)	Instruments	Main Results and Conclusions
**Basto-Pereira et al. (2016)**	To examine the impact of various forms of ACEs on juvenile justice involvement, criminal behaviour persistence, and psychosocial issues in young adulthood.	Portugal	*n* = 315	- ACE - BSI - EUROHIS-QOL-8 - D-CRIM	1. + adversity and negative outcomes such as juvenile justice involvement, criminal persistence implies + psychosocial problems during early adulthood. 2. CSA is the strongest predictor of juvenile justice involvement and criminal persistence predicted psychosocial problems.
**Basto-Pereira et al. (2022)**	Investigated the correlation between ACEs and delinquent conduct among young adults residing in ten countries spanning five continents, considering gender, age, and cross-cultural disparities.	Portugal, Spain, France, Mozambique, South Africa, Brazil, Iraq, Palestine, Thailand, and Australia	*n* = 3797 *M*_age_ = 18.97 *SD* = 0.81	- HDI - ACE - CVI	1. The country of residence has a significant association with self-reported criminal variety CPA, CSA, PN, and household substance abuse during the first 18 years of life are predictors of criminal variety among young adults aged 18–20 in both HDI top and bottom tier countries. 2. The strongest predictor of criminal variety varied by gender and HDI ranking, with CSA being the strongest predictor for females and those in HDI top-tier countries, and physical abuse being the strongest predictor for males and those in HDI bottom-tier countries.
**Burke et al. (2022)**	Verify what is the relationship between childhood maltreatment and violent behaviour and to define if these effects are different for women and men.	USA	*n* = 29,718 *M*_age_ = 47.25	- Alcohol Use Disorder and Associated - Disabilities Interview Schedule-5 - CTQ - CTS	1. Women who have experienced ACEs have + risk for engaging in violent behaviour. 2. + ACEs implies + violent criminal behaviour. 3. Adults who have been exposed to ACEs have + emotional dysregulation and self-regulation deficits,—decision making, and + behavioural problems. 4. Childhood trauma can significantly hinder the development of executive function and ↑ violent, aggressive, or criminal behaviour.
**Carvalho et al. (2019)**	To investigate the influence of physical, sexual, and emotional abuse, as well as physical and emotional neglect experienced during childhood and adolescence, on (a) domains of psychopathic traits and (b) altruistic attitudes during early adulthood.	Portugal	*n* = 673 *M*_age_ = 18.90 *SD* = 0.82	- ACE - YPI-S - Altruistic Attitudes Scale - Social and Family Situation Questionnaire;	1. CPA is associated with the behavioural dimension of psychopathy. 2. CEA is linked with the callous/unemotional traits of psychopathy. 3. + ACEs implies + juvenile delinquency and adult crime. 4. CSA is associated with different dimensions of youth psychopathy, including traits of manipulation, dishonest charm, callousness, and unemotionality. 5. Abuse and neglect have a negative correlation with altruistic attitudes. 6. EN is particularly important in inhibiting the development of normative altruism, impacting both the affective and behavioural dimensions of altruistic attitudes. 7. + ACEs implies the ↑ of possessing altruistic attitudes and ↑ the exhibition of psychopathic traits in young adults.
**Chopin et al. (2022)**	To investigate how childhood victimisation may contribute to the formation of risk factors that heighten the probability of engaging in online sexual misconduct.	Canada	*n* = 199	- Interview about childhood victimisation and polyvictimisation of the sample.	1. + ACEs implies + criminogenic cognitions, antisocial behaviours, and sexual interests in children. The specific type of victimisation experienced also plays a role in the likelihood of these outcomes. 2. + childhood abuse implies + offence-supportive cognitions, substance abuse, and youth engagement in sexual offending among individuals involved in online sexual offending during adulthood. 3. + Childhood trauma implies + sexual interests in children and a sense of loneliness.
**DeLisi et al. (2019)**	Investigate background factors preceding adverse childhood experiences and childhood psychopathology.	USA	*n* = 863 *M*_age_ = 44.00	- ACE - Psychiatric Diagnoses	1. The association between ACEs and symptoms of ASPD is not significant when ACEs are summed, but a significant association is observed with the diagnostic history of the disorder. 2. The familial progression of ASPD and ACEs are linked to the early family environments of criminal offenders’ environments.
**Donadio et al. (2021)**	To investigate how ACEs predict adult attachment styles, alcohol use, and relationship status in formerly incarcerated Black and Latino men.	USA	*n* = 248	- ECR-R - AUDIT - TEC	1. Formerly incarcerated Black and Latino adult men are at a high risk of experiencing disrupted attachments, as incarcerated Black and Latino adult men have + risk of experiencing disrupted attachments. 2. + ACEs implies + probability of development anxious and avoidant insecure attachment styles in adulthood. 3. ACEs predict insecure attachment patterns in formerly incarcerated Black and Latino men.
**Drury et al. (2019)**	Investigate the hypothesis that SO is associated with CSA was tested in the current study.	USA	*n* = 863 *M*_age_ = 43.71 *SD* = 11.45	- ACE	1. + CSA implies future sexual offending.
**Duin et al. (2020)**	To enhance the understanding of how CH and ACEs can predict the functioning of young adults facing severe problems in various areas of life.	Netherlands	*n* = 696 *M*_age_ = 22.00 *SD* = 2.40	- CTQ-SF - MANSA	1. + ACEs implies worst QoL outcomes. 2. + ACEs and criminal history imply problems in social functioning of a young adult.
**Edalati et al. (2020)**	To determine whether the relationship between an individual’s cumulative ACE score and the probability of experiencing CJI and victimisation remains significant over time, even after receiving the HF intervention.	Canada	*n* = 1888 *M*_age_ = 41.00 *SD* = 11.00	- ACE - HSJSU	1. + cumulative ACE score implies + victimisation; + ACE scores are more likely to experience victimisation than to be involved in the criminal justice system. 2. The relationship between cumulative ACE score, victimisation, and CJI remained significant, regardless of whether the participants were in the HF or TAU. 3. Cumulative ACE score did not moderate the effects of the intervention.
**Hill and Nathan (2008)**	Find if it is uncertain whether the origins of severe violence in childhood differ depending on the circumstances in which the violence takes place.	UK	*n* = 54 *M*_age_ = 29.16 *SD* = 5.74	- Interview Self-report violence in adulthood. - Interview Officially recorded adult offending. - Interview Adult antisocial personality disorder. - Interview Childhood maltreatment. - Interview Childhood psychopathology.	1. + variability in violence, in violent offenders, implies + diagnoses of adult ASPD and + childhood conduct disorder. 2. + interparental violence implies + social violence and partner violence.
**Hilton et al. (2019)**	Verify the association between ACEs and criminal tendencies among IPV offenders.	Canada	*n* = 435 men *M*_age_ = 34.31 *SD* = 12.51	- ACE - Cornier–Lang Criminal History Score - PCL-R - VRAG	1. + ACEs implies + criminal propensity in all models except institutional assaults. 2. Among IPV offenders, ACEs implies risk of violent recidivism, due to the overlap of parental alcoholism and separation from parents. 3. No significant effect of IPV on ACEs or criminal propensity measures, except for a lower risk of violent recidivism associated with having an intimate relationship history.
**James et al. (2020)**	Determining whether ACEs with psychopathology relevant to physical and sexual violence, such as psychopathy and sexual sadism, can predict the type of aggression displayed in the commission of a sexual homicide.	France	*n* = 120 *M*_age_ = 30.30 *SD* = 10.90	- ACE - PCL - SeSaS	1. ACEs during 0 and 12 years implies + sexual homicide using reactive aggression. 2. ACEs implies + reactive aggression in sexual murderers. 3. Inadequate parental behaviour implies + reactive aggression, manifesting impulsive or anger-driven homicides in the sample.
**Jones et al. (2018)**	Using a feminist life course theory approach, investigate the associations between individual, cumulative, and clusters of ACEs and multiple forms of IPV in adulthood.	USA	*n* = 355 *M*_age_ = 36.60	- CTS-R - PMWI - ACE	1. + ACEs ↑ the likelihood of experiencing multiple forms of IPV. 2. + CSA ↑ the risk of experiencing adult physical, sexual, and psychological abuse. 3. + CN in women ↑ the likely to report adult sexual abuse. 4. + CSA and neglect predict IPV and + severe physical injury in adult abusive relationships. 5. Women with 5 or more ACEs ↑ the likely to report adult physical, sexual, and psychological abuse compared to those who had fewer ACEs (4 or fewer). 6. + ACEs and IPV in the pre-prison lives of women prisoners, which suggests that childhood abuse, neglect, and chaotic home environments are linked to IPV in adulthood, indicating that ACEs and IPV are significant risk factors for women’s offending and incarceration.
**Jones et al. (2020)**	Apply a feminist life course theoretical framework in analysing the correlation between individual, cumulative, and clusters of ACEs and the perpetration of violence in intimate adult relationships of incarcerated women.	USA and non-American native	*n* = 356	- CTS2 - Self-report questionnaire - ACE	1. Women prisoners + ACEs more likely to perpetrate physical violence against an adult intimate partner. 2. The abuse cluster of ACEs ↑ with physical violence; −neglect ACE + related to violence against an intimate partner for Native American women prisoners. 3. A chaotic home environment was associated with violence for non-Native American women prisoners.
**Koolen and Vos (2022)**	Investigating the correlation between ACEs and personality disorders among individuals with antisocial, borderline, or narcissistic personality disorders who have a HO.	Netherlands	*n* = 102 *M*_age_ = 38.30 *SD* = 9.90	- CTQ - SMI-R - SNAP-F	1. ACEs did not differentiate between personality disorders. 2. + CPA and CEA explain 41% of the variance of BPD. 3. PN predict NPD, explaining 33.1% of the variance.
**Korsten and Vos (2022)**	The significance of childhood neglect in the treatment of male inpatients convicted of violent offenses is underscored by this study. Additionally, the study adds to our comprehension of the notion of therapy alliance among individuals with criminal convictions.	Netherlands	*n* = 99 *M*_age_ = 37.70 *SD* = 9.90	- WAI - SNAP - PCL-R	1. Therapy alliance is related to treatment outcome, recidivism, and adherence to probation conditions. 2. Childhood neglect is prevalent in offender populations.
**Lee et al. (2002)**	To determine the general, shared, and distinct developmental risk factors associated with paedophilia, exhibitionism, rape, and multiple paraphilias.	Australia	*n* = 97	- Marlowe–Crowne Social Desirability Scale - Parental Acceptance-Rejection Questionnaire - Parental Bonding Instrument - Family Adaptability and Cohesion Scale II - Youth Self-Report - Sexual Abuse Scale - Physical Abuse Scale	1. + CEA, family dysfunction, childhood behaviour problems, and CSA implies the ↑ of risk factors for paraphilias. 2. + CEA and family dysfunction implies the ↑ of risk factors for paedophilia, exhibitionism, rape, or multiple paraphilias. 3. + CSA implies the ↑ of risk factor for paedophilia.
**Levenson and Socia 2015)**	To investigate how ACEs affect arrest patterns in a sample of sexual offenders.	USA	*n* = 740	- ACE - Interview about adult health, mental health, and behavioural outcomes. - Questions about the characteristics of the sex offense committed.	1. ACEs ↑ the risk of criminal behaviour problems in adulthood for sex offenders. These offenders had + ACEs indicating exposure to various childhood maltreatments and chaotic households. 2. + ACE scores were associated with + assortment of arrest items, that ↑ the likelihood of versatile criminal behaviour. 3. The largest effect of ACE score on the arrest scale item is for nonsexual assault. 4. CSA, EN, and domestic violence in the childhood home were predictors of the total number of sex crime arrests, but not for non-sexual arrests, total arrests, or criminal versatility. CSA rarely occurs in isolation and often overlaps with other negative childhood experiences.
**Marotta (2021)**	Examine the correlation between occurrences of physical and sexual violence in childhood, adulthood, and both phases, and the RR of engaging in violent, sexual, and violent/sexual offenses.	USA	*n* = 13,606 *M*_age_ = 35.3 *SD* = 0.09	- Interview about sexual and non-sexual violent offending, experiencing sexual and non-sexual physical abuse during childhood and adulthood, household dysfunction.	1. + CPA both in adulthood and childhood are associated with ↑ RR of violent crime. 2. + CPA implies + violent crime perpetration but not sexual crime perpetration. 3. CSA is 3 times greater among people convicted of sexual offenses (15.3%) compared to those convicted of violence only (4.1%) and more than 5 times greater than those convicted of all other crimes (2.9%). 4. ACEs were associated with ↑ risk of being convicted for a violent crime.
**Moore and Tatman (2016)**	The extent to which ACE scores could serve as a predictor of re-offense risk in convicted offenders was examined.	USA	*n* = 141 *M*_age_ = 33.99 *SD* = 10.40	- ACE - LSI-R	1. + ACE scores predict future incarceration, recidivism, violent behaviour, and substance abuse. 2. + ACE scores implies poor physical and mental health, chronic disease, premature mortality, and functional limitations. 3. + ACE scores implies + scores on the LSI-R.
**Stinson et al. (2016)**	To investigates the influence of developmental challenges in early life on the emergence of aggressive and criminal conduct, as well as psychiatric hospitalisation for mental illness, among a sample of individuals in forensic mental health.	USA	*n* = 381 *M*_age_ = 43.00 *SD* = 12.26	- The variables were taken from the Social Service Reports, Psychiatric diagnosis and related symptoms and Social Service Records, being coded as present (1) and absent (0), except for the out-of-home foster care placements where absent is coded as 2.	1. Foster care placements affect a child’s development and attachment to caregivers. 2. Early trauma and foster care placement implies + negatives outcomes.
**Voith et al. (2017)**	Explore how CPA, CSA, and CEA are related to interpersonal violence in adulthood, specifically physical, sexual, and psychological victimisation and perpetration.	USA	*n* = 423 *M*_age_ = 22.00 *SD* = 5.30	- CTS 2 - SES-SFV - SES-SFP - CTQ - SDS	1. + CPA, in boys, implies + psychological violence against their partners and report multiple forms of perpetration. 2. + CPA, in men, implies the ↑ of risk of sexual and psychological victimisation by intimate partners and the ↑ to report polyvictimisation. 3. + CSA, in men, led to increased likelihood of perpetrating physical and sexual violence against their partners, using multiple forms of violence, and being likely to be victims of physical IPV and sexual violence, where alcohol use was mediator. 4. + CEA, in men, implies the ↑ the odds of experiencing physical and psychological victimisation in their intimate relationships and ↑ likelihood to report clinically depressive symptoms in early adulthood. 5. ACEs ↑ the risk of victimisation and perpetration in adulthood, but do not determine it.

Note. **ACE:** Adverse Childhood Experience Questionnaire; **ACEs:** adverse childhood experiences; **ASPD:** antisocial personality disorder; **AUDIT:** Alcohol Use Disorders Identification Test; **BPD:** borderline personality disorder; **BSI:** brief symptom inventory; **CEA:** childhood emotional abuse; **CH:** criminal history; **CJI:** criminal justice involvement; **CN:** childhood neglect; **CPA:** childhood physical abuse; **CSA:** childhood sexual abuse; **CTQ:** childhood trauma questionnaire; **CTQ-SF:** Childhood Trauma Questionnaire-Short form; **CTS 2:** Revised Conflict Tactics Scale; **CTS:** Conflict Tactics Scale; **CTS-R:** Revised Conflict Tactics Scale; **CVI:** Criminal Variety Index; **D-CRIM:** Self-report Questionnaire for measuring Delinquency and Crime; **ECR-R:** Experiences in Close Relationships-Revised; **EN:** emotional neglect; **HDI:** Human Development Index; **HF:** housing first; **HO:** History of Offending; **HSJSU:** Health Social, and Justice Service Use Inventory; **IPV:** intimate partner violence; **LSI-R:** Level of Service Inventory-Revised; **MANSA:** Manchester Short Assessment of Quality of Life; **NPD:** narcissistic personality disorder; **PCL:** Psychopathy Checklist; **PCL-R:** Psychopathy Checklist-Revised; **PMWI:** Psychological Maltreatment of Women Inventory; **PN:** physical neglect; **QoL:** quality of life; **RR:** relative risk; **SDS:** Marlowe–Crowne Social Desirability Scale; **SeSaS:** Severe Sexual Sadism Scale; **SES-SFP:** Sexual Experiences Survey-Short Form Perpetration; **SES-SFV:** Sexual Experiences Survey-Short Form Victimisation; **SMI-R:** Short version Schema Mode Inventory; **SNAP:** Schedule for Nonadaptive Personality **SNAP-F:** Schedule for Nonadaptive and Adaptive Personality; **SO:** sexual offending; **TAU:** treatment as usual; **TEC:** Traumatic Experience Checklist; **UK:** United Kingdom; **USA:** United States of America; **VRAG:** Violence Risk Appraisal Guide; **WAI:** working alliance inventory; **YPI-S:** Youth Psychopathic Inventory-Short Version. **+**: more: **–**: less; **↑**: higher.

**Table 2:** Association between ACEs and social, partner, and family violence.

	Social Violence	Partner Violence	Family Violence
Conduct disorder	Mean = 1.92, SD = 1.38, *p* < 0.001	Mean = 0.55, SD = 0.65, *p* = 0.78	Mean = 0.74, SD = 0.89, *p* = 0.50
Neglect	Mean = 2.23, SD = 1.42, *p* = 0.041	Mean = 0.69, SD = 0.75, *p* = 0.32	Mean = 0.62, SD = 0.65, *p* = 0.74
Physical abuse	Mean = 1.58, SD = 1.36, *p* = 0.99	Mean = 0.69, SD = 0.68, *p* = 0.009	Mean = 0.74, SD = 0.82, *p* = 0.51
Sexual abuse	Mean = 2.25, SD = 1.49, *p* = 0.12	Mean = 0.75, SD = 0.71, *p* = 0.31	Mean = 1.00, SD = 0.93, *p* = 0.27
Parental tension	Mean = 2.26, SD = 1.39, *p* = 0.001	Mean = 0.74, SD = 0.69, *p* = 0.043	Mean = 0.96, SD = 0.88, *p* = 0.046
Parental violence	Mean = 2.40, SD = 1.35, *p* = 0.004	Mean = 1.00, SD = 0.65, *p* = 0.001	Mean = 1.13, SD = 0.92, *p* = 0.017

**Table 3:** ACEs predicting sex crime arrests, non-sexual arrests, total arrests, and criminal versatility.

	Sex Crime Arrests	Non-Sexual Arrests	Total Arrests	Criminal Versatility
Verbal abuse	B = −0.118, SE = 0.119, β = −0.053 and Significance = 0.324	B = 0.306, SE = 0.165, β = 0.095 and Significance = 0.065	B = 0.167, SE = 0.222, β = 0.038 and Significance = 0.452	B = 0.165, SE = 0.161, β = 0.053 and Significance = 0.307
Physical abuse	B = −0.017, SE = 0.121, β = −0.008 and Significance = 0.886	B = −0.051, SE = 0.168, β = −0.016 and Significance = 0.761	B = −0.059, SE = 0.226, β = −0.013 and Significance = 0.795	B = 0.110, SE = 0.164, β = 0.035 and Significance = 0.504
Child sexual abuse	B = 0.207, SE = 0.096, β = 0.090 and Significance = 0.032	B = −0.071, SE = 0.134, β = −0.022 and Significance = 0.596	B = 0.124, SE = 0.181, β = 0.028 and Significance = 0.494	B = −0.096, SE = 0.132, β = −0.030 and Significance = 0.467
Emotional neglect	B = 0.305, SE = 0.104, β = 0.132 and Significance = 0.004	B = 0.007, SE = 0.146, β = 0.002 and Significance = 0.960	B = 0.296, SE = 0.196, β = 0.066 and Significance = 0.131	B = 0.044, SE = 0.141, β = 0.014 and Significance = 0.753
Physical neglect	B = −0.170, SE = 0.136, β = −0.055 and Significance = 0.214	B = 0.027, SE = 0.191, β = 0.006 and Significance = 0.887	B = −0.140, SE = 0.257, β = −0.023 and Significance = 0.585	B = −0.106, SE = 0.187, β = −0.024 and Significance = 0.572
Parents not married	B = −0.036, SE = 0.093, β = −0.016 and Significance = 0.695	B = 0.352, SE = 0.130, β = 0.110 and Significance = 0.007	B = 0.347, SE = 0.174, β = 0.080 and Significance = 0.047	B = 0.164, SE = 0.127, β = 0.053 and Significance = 0.197
Domestic violence in the home	B = 0.287, SE = 0.116, β = 0.110 and Significance = 0.014	B = 0.021, SE = 0.163, β = 0.006 and Significance = 0.897	B = 0.297, SE = 0.219, β = 0.058 and Significance = 0.176	B = 0.079, SE = 0.160, β = 0.022 and Significance = 0.621
Substance abuse in the home	B = 0.091, SE = 0.098, β = 0.041 and Significance = 0.354	B = 0.344, SE = 0.137, β = 0.107 and Significance = 0.013	B = 0.418, SE = 0.185, β = 0.096 and Significance = 0.024	B = 0.567, SE = 0.134, β = 0.183 and Significance = 0.000
Mental illness in the home	B = 0.006, SE = 0.108, β = 0.003 and Significance = 0.952	B = 0.229, SE = 0.151, β = 0.063 and Significance = 0.130	B = 0.241, SE = 0.202, β = 0.049 and Significance = 0.234	B = 0.221, SE = 0.148, β = 0.062 and Significance = 0.136
Incarceration of a family member	B = 0.344, SE = 0.109, β = 0.129 and Significance = 0.002	B = 0.800, SE = 0.152, β = 0.209 and Significance = 0.000	B = 1.166, SE = 0.205, β = 0.224 and Significance = 0.000	B = 0.450, SE = 0.149, β = 0.121 and Significance = 0.003
